# Obesity and Diabetes: A Sword of Damocles for Future Generations

**DOI:** 10.3390/biomedicines8110478

**Published:** 2020-11-06

**Authors:** Alessia Angi, Francesco Chiarelli

**Affiliations:** Department of Pediatrics, University of Chieti, 66100 Chieti, Italy; chiarelli@unich.it

**Keywords:** obesity, diabetes, childhood, atherosclerosis, cardiovascular disease

## Abstract

Childhood obesity is one of the most challenging problem of the 21st century. The prevalence has increased, reaching an alarming rate. Furthermore, the problem is global and is also affecting low- and middle-income countries. This global obesity epidemic explains how the roots of cardiovascular disease, the most common cause of mortality among adults, begin in childhood. Overweight and obese children are likely to stay obese into adulthood and to develop noncommunicable diseases such as diabetes and cardiovascular diseases at a younger age. Thus, prevention should be the major goal and should start early in life. The aim of this review is to present an updated framework of the current understanding of the cardiovascular and metabolic risks in obese children and adolescents and to discuss the available therapeutic options.

## 1. Introduction

Obesity is a multifactorial disease and various genetic, behavioural and sociocultural characteristics can affect its development [1]. The prevalence of obesity in children and youth has reached dramatic dimensions globally and still remains one of the most challenging problems in developed countries [2]. The Global Burden of Disease Study has presented data according to which the prevalence of childhood obesity has doubled in more than 70 countries since 1980 [3]. This global obesity epidemic explains how the roots of cardiovascular disease, the most common cause of mortality among adults, begin in childhood [4]. One predictor of future cardiovascular risk is the Metabolic Syndrome (MetS) [2], a cluster of cardiovascular risk factors including central obesity, hypertension, dyslipidemia and dysglicemia [5]. Furthermore, MetS also correlates with the risk of type 2 diabetes (T2D) [6]. Behavioural and therapeutic interventions have only shown modest success to date. Thus, the prevention of obesity should be the major goal and should start early in life.

## 2. Definition of Obesity

The World Health Organization (WHO) defines obesity as an excess in the fat mass that is great enough to increase the risk of morbidity, altered physical, psychological or social well-being, and/or mortality [7]. The body mass index (BMI) shows a good correlation with body fat percentage and is used as an index of relative weight [8]; as is well known, the BMI is calculated by dividing the body weight in kilograms by the height in square meters. In general, the BMI estimates adiposity in healthy children, but it may slightly overestimate fatness in children who are short or who have a relatively high muscle mass, and it may underestimate adiposity in children with reduced muscle mass [9]. The BMI of children changes with age in relation to growth, and, consequently, classification is based on the child BMI compared to the age and sex standard, expressed as the Z score [10]. BMI reference standards for children between two and 20 years of age were published in 2000 by the National Center for Health Statistics and the Center for Disease Control and Prevention (CDC) [11]. The WHO also defined BMI cut-offs for children under two years of age [12]. The CDC recommends the use of curves based on the WHO child growth standard for children and toddlers younger than two years and the CDC/National Center for Health Statistics growth references for children two years and older [11,13]. The ability for any of the various childhood BMI cut-offs to predict the presence of adult cardiovascular risk factors or disease remains largely unknown [14]. In some studies, the waist circumference (WC) and waist:height ratio (WHtR) have been shown to predict cardio-metabolic risk, correlating more strongly than BMI with several complications such as insulin resistance (IR), dyslipidemia and nonalcoholic fatty liver disease [15]. It would seem reasonable to include the WC and WHtR calculation in the routine evaluation of overweight and obese children in order to assess abdominal adiposity [14].

## 3. Epidemiology of Childhood and Adolescent Obesity

In recent decades, obesity in children and adolescents has emerged as a serious health issue worldwide [1]. The Global Burden of Disease Study has systematically evaluated the prevalence of childhood overweight and obesity since 1980 and has shown that obesity prevalence has doubled in more than 70 countries worldwide and has approximately tripled in some developing countries since then [16,17]. In particular, in developed countries the prevalence also increased from 16.9% in 1980 to 23.8% in 2013 for boys and from 16.2% in 1980 to 22.6% in 2013 for girls [3]. In developing countries, the prevalence of overweight and obesity reached 12.9% for boys and 13.4% for girls in 2013 compared with 8.1% for boys and 8.4% for girls in 1980 [3]. In 2015, a total of 107.7 million children (and 603.7 million adults) were classified as obese, corresponding to a worldwide prevalence of childhood overweight and obesity of 23% [16,18]. Furthermore, the prevalence of obesity varies by age: 8.4% of preschool children (age, 2–5 years), 17.7% of school-age children (age, 6–11 years) and 20.5% of adolescents (age, 12–18 years) have obesity in the United States of America (USA) [19]. Despite the recent plateau in the overall prevalence of childhood obesity in the USA, the prevalence of severe obesity (defined in this report as BMI ≥ 95th percentile or BMI ≥ 35 kg/m^2^) has continued to increase, leading to the emergence of multiple serious comorbidities [19,20]. Obesity-associated comorbidities are primarily related to cardio-metabolic disease and represent the most significant economic and social public health burden of the obesity epidemic [21]. In fact, in 2015 obesity accounted for about four millions deaths worldwide, and 70% were due to cardiovascular disease (CVD) [16,22]. Unfortunately, a high percentage of children with obesity carry their adiposity into adulthood, especially those with the most severe degrees of obesity and those in older age groups [13]. In addition, it has been described that 71% of adolescents with severe obesity continued to have severe obesity in adulthood compared with only 8% of adolescents with mild obesity [23]. As obesity tends to track into adulthood, it is mandatory that prevention and intervention strategies should begin at the earliest possible age [24].

## 4. Etiology of Obesity

On the basis of etiology, childhood obesity can be subdivided into exogenous and endogenous types [25]. A chronic imbalance between energy intake and expenditure is the cause of exogenous obesity; at variance, endogenous obesity is caused by genetic, syndromic and endocrine causes [25]. To date, children live in an increasingly obesogenic environment where gaining excessive weight is very easy [25]. Children have unhealthy dietary habits (e.g., the consumption of energy-dense food, sugar-sweetened beverages, poor intake of fruits and vegetables, excessive snacking) associated with a lack of physical activity, increased television viewing time and reduced sleep time. These obesogenic individual behaviours are linked to surrounding microenvironments, including family, schools and neighbourhoods and to macroenvironments like the food industries and government [26]. The Task Force for Pediatric Obesity of the Endocrine Society has described obesity-associated endocrine causes or syndromic conditions [27]. Thus, it is important to identify endocrine and syndromic causes in order to manage these specific conditions. Red flags for pathologic obesity that may warrant further investigation include a rapid onset of weight gain, very early age of onset, obesity discordant with parent weights, hypogonadism, short stature/poor linear growth, and the association of dysmorphic features or developmental delay [20]. Physicians should test for endocrine causes (e.g., hypothyroidism, Cushing syndrome, growth hormone deficiency) of obesity when there is evidence of stunted growth relative to the individual’s genetic potential and when there is a decreased growth velocity against the backdrop of a continued weight gain [28]. Among monogenic defects affecting the leptin-melanocortin regulating pathway, the most common are MCR4 mutations (affecting 4% of early onset and childhood severe obesity) [29]. Genetic syndromes generally show characteristic dysmorphic features and/or neurocognitive delay (e.g., Prader–Willi, Bardet–Biedl, WAGR syndrome) [30].

## 5. Obesity and Cardiovascular Risk

**Figure 1:**
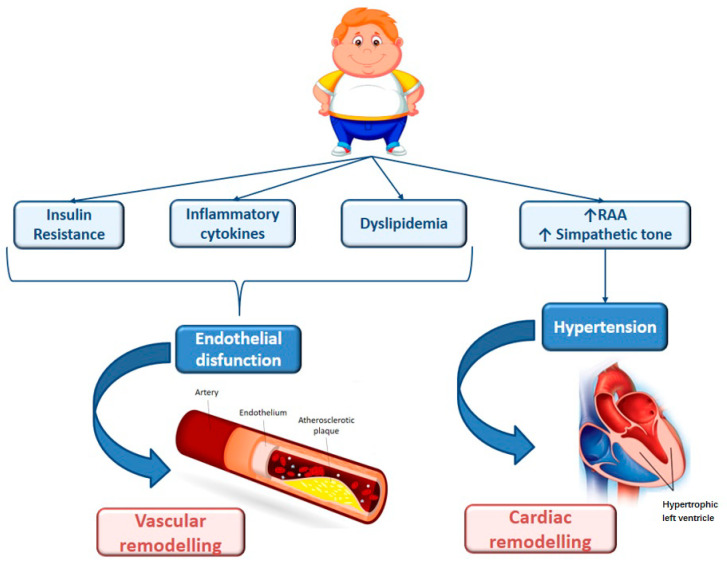
Risk factors for cardiovascular remodelling in obese children. The arrow means that there is an increase in sympathetic tone and in the activity of RAA system.

One of the important health implications of obesity in children includes the development of CVD risk factors during childhood and adolescence [31]. CVD is the leading cause of global mortality, accounting for 17.5 million deaths in 2005, and it is projected to rise to 23.6 million deaths by 2030 [32]. Although atherosclerosis manifests clinically in middle and late adulthood, it is well-known that it has a long asymptomatic phase of development, which begins early in life, often during childhood and adolescence [33]. Atherosclerosis begins in childhood with the so called “fatty-streaks” in the intima media of arteries, which may progress into fibrous plaque. The buildup of arterial plaques or their rupture often leads to symptoms of CVD such as angina, myocardial infarction and stroke [33,34]. The first signs of atherosclerosis are described as occurring starting from pregnancy. In fact, foetal studies documented the presence of fatty streak formation in human foetal arteries in over 50% of foetuses of hypercholesterolemic mothers [35]. Moreover, the Fate of early lesion in children (FELIC) study demonstrated that atherosclerosis in children of hypercholesterolemic mothers progresses much more rapidly than it did for children of mothers who had normal cholesterol levels [36]. It seems that foetal lesion formation is associated with genetic programming, which may in turn affect postnatal atherogenesis, supporting the important role of epigenetics in this mechanism [37]. Furthermore, observational studies from autopsies have helped to define the timing, extent and severity of atherosclerotic lesions. In particular, fatty streaks are present from early childhood and are well-established by 20 or 30 years of age, while raised plaques increase in terms of prevalence and extent during the 15–34 year age span [38]. The Bogalusa Heart Study examined the extent of fatty streaks and fibrous plaques in the aorta and coronary arteries of 204 patients (aged 2–39 years) [4]. The prevalence and the extent of atherosclerosis was greater with increasing age, BMI, blood pressure and levels of serum total cholesterol (TC) and low-density lipoprotein cholesterol (LDL-C). In a larger multicentre study of 2876 autopsied young persons aged 15–34 years, the Pathobiological Determinants of Atherosclerosis in Youth (PDAY), obesity was an independent and a prominent risk factor for fatty streaks [39]. The extent of atherosclerotic lesions was associated with a high level of LDL-C and low high-density cholesterol (HDL-C), hypertension, obesity and impaired glucose tolerance. In addition to autopsy findings, studies using noninvasive measures, including carotid intima media thickness (cIMT) and arterial distensibility, have shown anatomic and functional changes of atherosclerosis in youth. cIMT progresses with age and risk factors predict thickness in young adults [40]. The risk factors are the same as those associated with advanced lesions: increased BMI, hypertension, dyslipidemia, IR and cigarette smoking. However, it is important to discern the independent contribution of childhood obesity to CVD and diabetes. The International Childhood Cardiovascular Cohort consortium demonstrated that childhood obesity is a strong independent risk factor for arterial vascular abnormalities [41]. The pathological association of obesity and CVD may be explained by the so-called “adiposopathy”. Abdominal obesity may be the source of mediators, known as adipocytokines, that induce a condition of IR, systemic inflammation and sympathetic activation that, in turn, leads to vascular and cardiac remodelling [42] (Figure 1). This process may be reversible; children with obesity who transitioned to a normal weight status as adults had a risk comparable to persons who were never obese [43]. Therefore, recognizing this process during childhood is the key to adopting measures that will prevent atherosclerosis and eliminate future cardiovascular-related events [14].

## 6. Dysglycaemia and Insulin Resistance

Dysglycaemia is a term that includes impaired fasting glucose (IFG), impaired glucose tolerance (IGT) and diabetes [44]. The prevalence of IFG and IGT in adolescents with obesity was 16.8% and 6.6%, respectively [45]. The childhood obesity epidemic is associated with a three-fold increase in the prevalence rates of type 2 diabetes in adolescents and young adults over the last three decades [21]. Using a two-hour glucose threshold of more than 140 mg/dL, up to 21% of obese youth are classified as having prediabetes, with a progression to diabetes of 10–15% per year [46]. The onset of T2D occurs mostly in adulthood, while among children it develops during the second decade of life and in middle to late puberty [47]. The pathogenesis of T2D is linked to obesity combined with insulin deficiency [48]. Global adiposity acts as the most important culprit of IR, which appears early in the disease and is primarily compensated by hyperinsulinemia [47]. The relation between obesity and IR is weaker in African American children because of a higher obesity-independent hyperinsulinemia and a poor β-cell adaptation to IR [49,50]. The mechanisms responsible for the development of IR in obese children are not completely understood [51]. Fatty acids, inflammatory cytokines and growth factor are included in the pathogenesis [52,53,54]. Because of the absence of a standardized method to measure insulin sensitivity, an unanimous definition of IR does not exist [51]. IR could be defined as a condition in which greater concentrations of insulin are needed to determine a physiological effect that was previously induced by a lower concentration [55]. The gold standard technique used to evaluate IR is the hyperinsulinemic-euglycemic clamp; however, it is costly and difficult to perform in clinical and research sets [56]. Therefore, several surrogate markers have been proposed, such as the Matsuda index [57] and Homeostatic Model of Assessment-insulin resistance (HOMA-IR) [58]. However, it was proven that the distribution of fat tissue was the crucial factor for developing IR [59]. Children with a high proportion of visceral fat are more insulin-resistant and have a higher plasma glucose in the second hour of the glucose tolerance test [48,60]. The role of visceral adiposity is particularly determinative in pubertal and postpubertal obese children and adolescents, whereas it is a minor determinant of IR in prepubertal children [61]. In adolescents, it is important to highlight that physiological hormonal modifications are responsible for a transient reduction in the whole body insulin sensitivity, which may resolve at the end of puberty [62,63]. Furthermore, it is important to highlight the “obesity paradox”. Patients with a normal weight at the time of diagnosis of T2D had a higher risk of developing a cardiovascular complication, at variance of people with a higher weight during the onset of diabetes [64]. Diabetes can lead to many complications (CVD, nephropathy, retinopathy and microangiopathy), and obesity in children with T2D increases the risk of cardiovascular complications like myocardial infarction, stroke and renal failure. The cardiovascular risk in youth is related both to comorbidities (dyslipidemia, hypertension, nonalcoholic steatosis) and to the hyperglycaemic effect on the vasculature [21].

Disglycaemia and elevated BMI in children are determinants of adverse arterial wall adaptation through the formation of advanced glycation end products, which can lead to coronary artery calcification and increased cIMT [65]. The rapid nature of T2D in children accelerates the progression of micro- and macrovascular complications, but short-term improvement in glycaemia did not decrease the prevalence of cardio-metabolic risk factors [66]. The risk of microvascular disease and death from cardiovascular disease occurs 10–15 years after the onset of the disease [67,68]. Further studies are needed to identify target therapies to reduce this risk [21].

## 7. Dyslipidemia

Obese children and adolescents have been observed to have a more unfavourable lipid profile than children and adolescents with a normal body weight [69]. The atherogenic lipid pattern associated with childhood obesity consists of a combination of elevated triglycerides (TG), decreased HDL-C and top normal to mildly elevated LDL-C [70]. Although the pathogenetic mechanism of dyslipidemia is multifactorial and still debated, IR has been hypothesized to play a major role in the relationship between dyslipidemia and insulin obesity [71,72]. Some authors observed that children with a greater degree of IR had a higher risk of developing atherogenic dyslipidemia than those with a moderate IR status [72]. Moreover, it has been demonstrated that the TG/HDL ratio is significantly associated with insulin and early signs of vascular damage in obese children [73,74].

The cause of atherogenesis mainly remains the subendothelial retention of LDL-C containing lipoprotein and the decrease in HDL-C particles [75,76]. Small dense LDL-C (SdLDL) show an increased susceptibility to oxidation, thus promoting endothelial damage and infiltrating the arterial wall [77]. The prevalence of SdLDL correlates with IR and visceral adiposity and shows a strong correlation with the common cIMT [78]. In fact, the atherogenicity of the combined dyslipidemia seen with childhood obesity manifests in structural and functional vascular changes assessed noninvasively as increased cIMT and increased arterial stiffness [79]. The longitudinal Young Finns study revealed that, at 21-year follow-up, subjects with a combined dyslipidemia pattern beginning in childhood had significantly increased cIMT compared with normolipemic controls, even after adjustment for other risk factors [80]. In adults, combined dyslipidemia is the most prevalent pattern seen in individuals presenting with early clinical cardiovascular events [81]. Thus, the combined dyslipidemia pattern seen with obesity in childhood is increasing in prevalence and predicts vascular dysfunction in young adulthood and early clinical events in adult life [82].

## 8. Hypertension

Hypertension in childhood is defined as either a systolic or diastolic blood pressure value greater than or equal to the 95th percentile for sex, age and height on at least three occasions [83]. Historically, childhood hypertension has been considered a rare condition, secondary to renal, cardiac or endocrine disorders. However, the prevalence of primary hypertension in children and youth has increased in parallel with the growing prevalence of overweight and obesity. In fact, to date, 25% of obese children and adolescents suffer from hypertension [84]. In particular, some authors found that each increase by 10 BMI units was associated with an increase in the systolic blood pressure of 10 mmHg and in the diastolic blood pressure of 3 mmHg [85]. Furthermore, the risk remains as children with obesity progress into adulthood [86].

The pathophysiology of hypertension includes the activation of the sympathetic nervous system and of the renin-angiotensin-aldosterone system (RAAS), which increase the intravascular volume and consequently the ventricular preload [84]. Proinflammatory cytokines and oxidative stress contribute to vascular endothelial dysfunction, an impaired local vasodilator response and increased peripheral resistance, particularly with vascular afterload [85]. These changes may induce alterations and remodelling of large vessels and the heart [87]. Some authors described the fact that obese children had a larger left ventricular mass index and an earlier cardiac impairment (systolic and diastolic dysfunctions) compared to healthy controls [88,89]. Furthermore, repetitive obstructive apneas/hypopneas cause dramatic fluctuations in intrathoracic pressure and blood pressure, increased left ventricular preload, left atrial dilatation, arrhythmias (particularly atrial fibrillation), and left ventricular eccentric hypertrophy [90]. The increased ventricular strain persists into adulthood and raises the risk of CVD, but fortunately it is reversible with the normalization of weight [87]. Thus, a timely diagnosis and initiation of treatment are important to reduce the risk of end-organ damage [85].

## 9. Liver Steatosis

Nonalcoholic fatty liver disease (NAFLD) is a chronic liver disorder that is increasingly prevalent with the worldwide epidemic of obesity. The term NAFLD describes a spectrum of liver pathology ranges from simple steatosis to nonalcoholic steatohepatitis and even cirrhosis [91]. Pediatric obesity increases the risk of hepatic steatosis and steatohepatitis, so that NAFLD occurs in 34% of youth with obesity [92]. The most credited model for the pathogenesis of NAFLD is the ‘‘two-hit’’ theory, where the first hit is represented by the IR, responsible for the abnormalities in lipid storage and lipolysis, which therefore leads to an increased fatty acids flux from adipose tissue to the liver and to the subsequent accumulation of TG into the hepatocytes [93]. The “second hit” might be represented by the oxidative stress, which activates the inflammatory cascade and generates reactive oxygen species such as hydroxyl radicals and superoxide anions, which react with the excess lipid to form peroxides [94,95]. A large body of emerging literature seems to suggest that intestinal microbiota is also involved in the development of obesity and its complications, including obesity-related liver disease [91]. The diagnosis can be difficult and requires a liver biopsy, but elevated aminotransferase in obese children should prompt the clinician to investigate for NAFLD [96].

## 10. Metabolic Syndrome

Obesity plays a key role in the development of MetS, a complex picture characterized by a combination of risk factors, such as WC, TG, HDL-C, BP and glucose [87].

Data from multiracial cohorts of children have shown that the degree of obesity and the prevalence of MetS are strongly associated [97]. There is currently no consensus guideline for the diagnostic criteria for paediatric MetS [2]. In fact, more than 40 definitions have been reported in children, and most authors have adapted criteria from the adult population [98] (Table 1). The American Heart Association (AHA) adapts the adult definition to the paediatric population [99]. In 2007, the International Diabetes Federation (IDF) provided a definition of MetS for children aged 10–16 years, while adult criteria were still adopted for children older than 16 years [100]. The Identification and prevention of Dietary and lifestyle-induced health Effects In Children and infantS (IDEFICS) consortium recently provided a definition of paediatric Mets using percentiles for each diagnostic criterion [101]. There is a common agreement to include IR, central obesity, hypertension and dyslipidemia as major components of MetS [101]. However, there are certain limitations to the clinical use of this definition due to different applied criteria, heterogeneous cut-off values and missing values for prepubertal children. In addition, some authors propose to include other features in the definition of MetS, such as nonalcoholic fatty liver disease, hyperuricemia and sleep disturbance [51]. The prevalence of MetS in children and youth has been estimated to differ between 6 and 39% depending on which diagnostic criteria are applied [102]. In addition to the degree of obesity, fat distribution also appears to be important. Visceral fat accumulation, independent of BMI, has been shown to be strongly associated with both childhood MetS and CVD later in life [103].

The pathogenesis of MetS is not fully understood, but central obesity appears to be the central driver [5]. Visceral adipocytes release chemo-attractants and cytokines, leading to systemic inflammation [104]. Furthermore, the reduced production of adiponectin and the higher release of free fatty acids increase the oxidative stress [105]. This in turn reduces insulin’s ability to stimulate glucose transporters to the cell surface, causing IR [6].

## 11. Lifestyle Changes

Many studies have shown that the weight status in early childhood is a significant predictor for the weight status and associated cardio-metabolic comorbidities later in life [106,107]. Therefore, early intervention could influence future morbidity and mortality as well as improve the quality of life. The first approach to MetS is to try a lifestyle intervention with dietary modifications and increased physical activity [27]. These recommendations are difficult to achieve, especially for adolescents. Thus, the prevention or treatment of childhood obesity should start as early as possible. Due to the fact that therapeutic approaches often only show modest effects, prevention should be the primary goal. The WHO commission suggests individual and community based-prevention strategies to fight the obesity epidemic [108]. Physical activity is not only associated with weight loss but also has a beneficial effect on insulin sensitivity independently from adiposity [109]. The Endocrine Society Clinical Practice Guidelines suggest a minimum of 30 min of moderate to vigorous daily physical activity with a goal of 60 min daily [27]. A combination of low aerobic and resistance exercises is recommended because it seems to improve insulin sensitivity [110]. Furthermore, other health behaviours such as dietary intake and sleeping habits can affect insulin sensitivity. In particular, a short sleep duration (<9 h/day) and sleep apnea are linked to IR [111].

The optimal nutritional management is still debated. The main approach for dietary changes for children and adolescents, recommended by the American Academy of Pediatrics, the AHA and the WHO, is an increase in vegetable and fruit consumption and a reduced intake of saturated fat and sugar [112]. Several studies demonstrated that the consumption of food with a higher fibre intake was associated with a higher insulin sensitivity [113,114]. A diet with a higher fibre intake offers several beneficial effects, such as increased satiety, slowing the absorption of carbohydrates and the bulking effect of adding low-energy food to the diet [115]. Low glycaemic index foods decrease blood glucose and insulin excursion, promote fat oxidation and increase satiety, but their effect on IR is still unclear [116,117]. The most effective interventions are likely to include a combined approach incorporating dietary and physical activity changes [5]. This is due to the fact that an increased energy expenditure may lead to a compensatory increase in food intake [118], while isolated caloric restriction results in a lowering of the basal metabolic rate [119].

## 12. Pharmacotherapy

Medications are suggested for children and adolescents for whom a lifestyle management of obesity has not resulted in improvement [96]. To date, the only medications approved by the US Food and Drug Administration (FDA) are orlistat and phentermine for adolescents aged >12 years and >16 years, respectively [2]. Metformin has been approved to treat T2D in children 10 years or older, while its effect on weight loss or BMI reduction are rather limited [120]. However, its results show that it is a promising method to improve glucose metabolism and reduce cardio-metabolic risk [121]. Other potential pharmacologic options are glucagon-like peptide-1 (GLP-1) analogues such as liraglutide. They increase the postprandial insulin level, reduce glucagon secretion, delay gastric emptying and induce weight loss through reductions in appetite and energy intake [122]. However, they have been approved by the FDA in addition to lifestyle interventions for the treatment of obese and overweight adults with at least one weight-related coexisting condition [123]. Obese children with dyslipidemia should be treated with statin if a lifestyle modification fails or if LDL levels are higher than 160 mg/dl [27]. If obese children manifest with arterial hypertension, pharmacotherapy might be considered in the second line and should be started with an ACE inhibitor [124].

## 13. Conclusions

Obesity still remains one of the global burdens in medicine, particularly because of its high prevalence in children and adolescents and its associated cardio-metabolic sequelae [125]. As many obese children remain obese until adulthood and as the first signs of atherosclerosis start in childhood, an early normalization of body weight is becoming necessary and of paramount importance. Regardless of the etiology, all patients should be assessed for modifiable lifestyle risk factors and screened for the complications of obesity [25]. Initial management includes dietary and physical activity changes, while pharmacotherapy is recommended for a small minority of patients. However, intervention strategies have only shown a limited effect to date and cannot prevent long-term consequences [25]. Thus, prevention remains the best approach to halt and reverse the current epidemic of childhood obesity and should start as early in life as possible [2].

## Figures and Tables

**Table 1:** Definition of metabolic syndrome in children.

	Cook et al., 2003 (NCEP)	Weiss et al., 2004 (NCEP)	Zimmet et al., 2007 (IDF)	Ahrens et al., 2014 (IDEFICS)
WC	≥90° ptc		≥90° ptc	≥90°(95°) ptc
SBP	≥90° ptc	≥95° ptc	≥130 mmHg	≥90°(95°) ptc
DBP	≥90° ptc	≥95° ptc	≥85 mmHg	≥90°(95°) ptc
TG	≥1.24 mmol/L	≥95° ptc	≥1.7 mmol/L	≥90°(95°) ptc
HDL-C	≤1.03 mmol/L	≤5° ptc	≤1.03 mmol/L	≤10°(5°) ptc
Glucose homeostasis	IFG ≥ 6.11 mmol/L	IGT (ADA criteria)	IFG ≥ 5.6 mmol/L	HOMA-IR or IFG ≥90° (95°) ptc
BMI		Z score ≥ 2		

ptc: percentile; WC: waist circumference; SBP: systolic blood pressure; DBP: diastolic blood pressure; TG triglycerides, HDL-C: high-density lipoprotein cholesterol; BMI: body mass index; IFG: fasting glucose; IGT: impaired glucose tolerance; HOMA-IR: Homeostatic Model Assessment of Insulin Resistance; NCEP: National Cholesterol Education Program; IDF: International Diabetes Federation; IDEFICS: Identification and prevention of Dietary and lifestyle-induced health Effects In Children and infantS.

## Abbreviations

MetS	Metabolic Syndrome
WHO	World Health Organization
BMI	body mass index
CDC	Center for Disease Control and Prevention
WC	waist circumference
WHtR	waist-to-height ratio
CVD	cardiovascular disease
TC	total cholesterol
LDL-C	low-density lipoprotein cholesterol
HDL-C	high-density lipoprotein cholesterol
PDAY	Pathobiological Determinants of Atherosclerosis in Youth
cIMT	carotid intima media thickness
IR	insulin resistance
IFG	impaired fasting glucose
IGT	impaired glucose tolerance
T2D	type 2 diabetes
HOMA-IR	Homeostatic Model of Assessment-insulin resistance
SdLDL-C	Small dense low-density lipoprotein cholesterol
TG	triglycerides
RAAS	renin-angiotensin-aldosterone system
NAFLD	Nonalcoholic fatty liver disease
AHA	American Heart Association
IDF	International Diabetes Federation
IDEFICS	Identification and prevention of Dietary and lifestyle-induced health Effects In Children and infantS
FDA	Food and Drug Administration
USA	United States of America
